# Correction: Ionizing radiation induces ataxia telangiectasia mutated kinase (ATM)-mediated phosphorylation of LKB1/STK11 at Thr-366

**DOI:** 10.1042/BJ20021284_COR

**Published:** 2025-04-01

**Keywords:** ataxia telangiectasia-related kinase (ATR), DNA-dependent protein kinase (DNA-PK), Peutz-Jeghers syndrome, phosphopeptide mapping

It has come to the attention of the authors of the article “Ionizing radiation induces ataxia telangiectasia mutated kinase (ATM)-mediated phosphorylation of LKB1/STK11 at Thr-366” (DOI: 10.1042/bj20021284) that there was a partial duplication of Western blots in Figures 2 and 7, and also a duplication within Figure 2. These were unintentional errors made during the assembly of the Figure. Corrections have been provided for Figures 2 and 7, consistent with the raw data.

**Figure 7:**
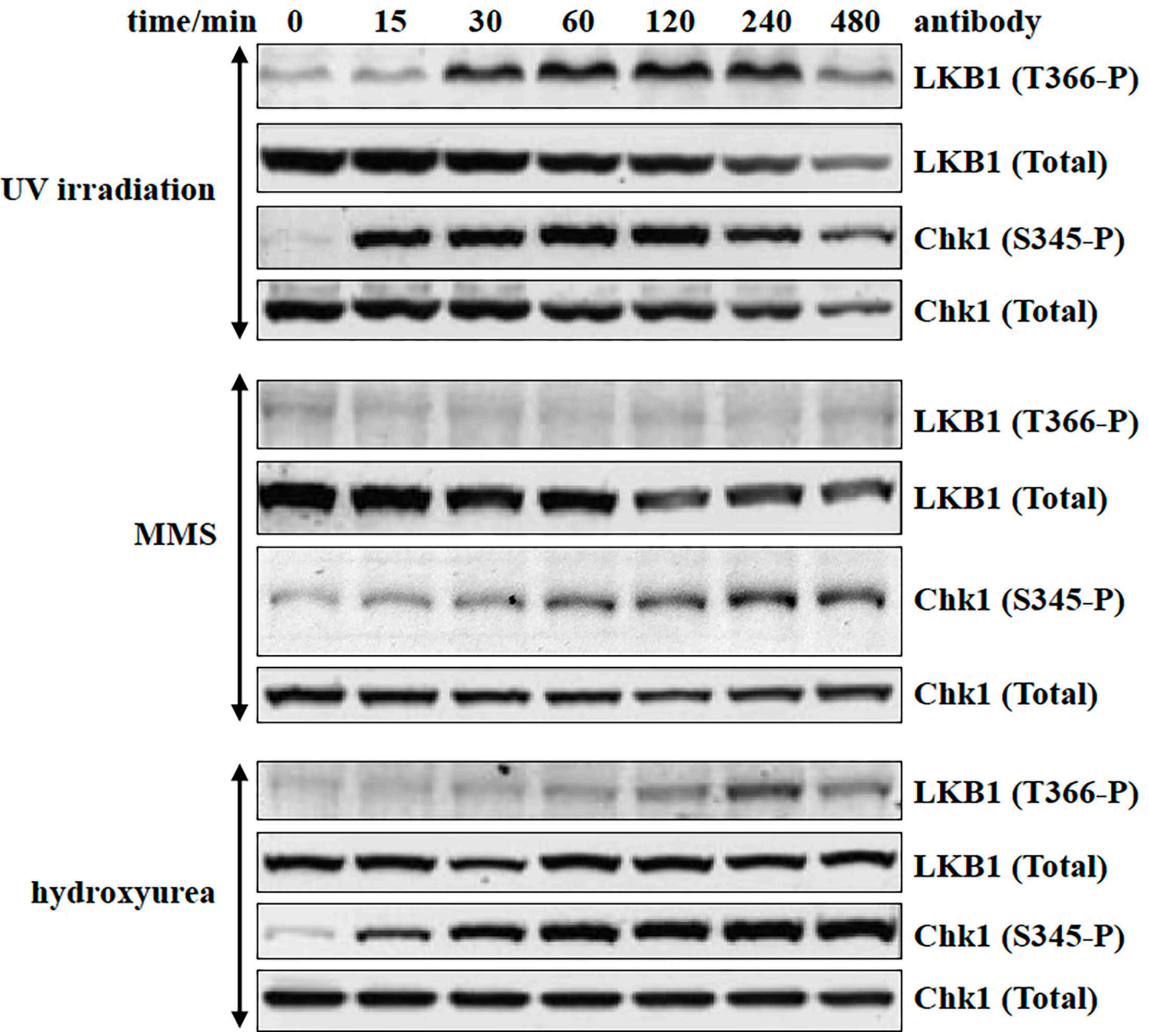
Evidence that LKB1 is a poor substrate for ATR. HeLa cells stably expressing WT-LKB1 were cultured with 1 µg/ml tetracycline for 24 h prior to lysis. The cells were exposed to UV (200 J/m2), MMS (0.01%, v/v) or hydroxyurea (2 mM) for the times indicated. Total cell lysate (20 µg) was immunoblotted with the indicated antibodies. Similar results were obtained in two (MMS and hydroxyurea) and four (UV) separate experiments.

**Figure 2:**
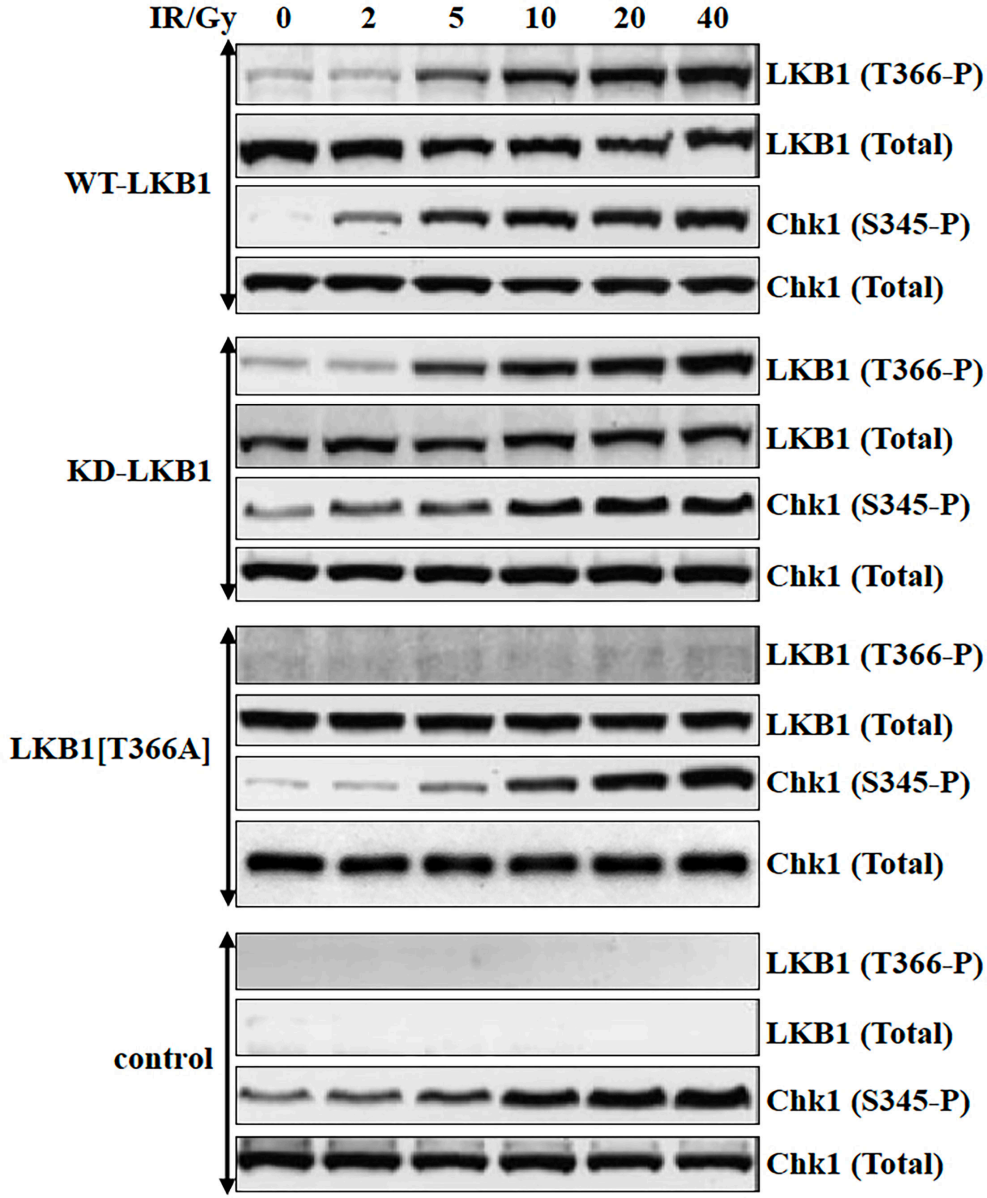
IR induces phosphorylation of KD-LKB1 at Thr-366. HeLa cells stably expressing WT-LKB1 (top panel), KD-LKB1 (second panel), LKB1[T366A] (third panel) or control HeLa cells transfected with empty pcDNA4/T0 vector (bottom panel) were cultured with 1 µg/ml tetracycline for 24 h prior to lysis. The cells were exposed to the indicated dose of IR and lysed after 30 min. Total cell lysate (20 µg) was immunoblotted with the indicated antibodies. Similar results were obtained in three separate experiments.

With the change in Figure 7, text on page 513 needs to be changed.

Original text: “The ATR activators MMS (Figure 7, middle panel) and hydroxyurea (Figure 7, bottom panel) failed to stimulate significant phosphorylation of LKB1 at Thr-366, whereas they markedly promoted Chk1 phosphorylation within 15 min.”

Revised text: “The ATR activators MMS (Figure 7, middle panel) and hydroxyurea (Figure 7, bottom panel) failed to stimulate significant phosphorylation of LKB1 at Thr-366, whereas they markedly promoted Chk1 phosphorylation in a time-dependent manner.”

The raw data and requested correction have been assessed by and agreed with the Publisher. The authors apologise for the errors and any inconvenience caused.

The corrected Figures 2 and 7 are presented here.

